# A severe case of *Trichophyton rubrum*-caused dermatomycosis exacerbated after COVID-19 vaccination that had to be differentiated from pustular psoriasis

**DOI:** 10.1016/j.mmcr.2022.03.001

**Published:** 2022-03-12

**Authors:** Yuta Norimatsu, Yurie Norimatsu

**Affiliations:** a Department of Dermatology, University of Tokyo Graduate School of Medicine, 113-8655, 7-3-1,Hongo, Bunkyo-ku, Tokyo, Japan; b Department of Dermatology, Ageo Daiichi Clinic for Internal Medicine and Pediatrics, 362-0064, 845-1 Koshikidani, Ageo, Saitama, Japan

**Keywords:** *Trichophyton rubrum*, dermatomycosis, Pustular psoriasis, Fosravuconazole, SARS-CoV-2 vaccination, BNT162b2

## Abstract

We present a case of deep dermatomycosis caused by *Trichophyton rubrum* that developed after administration of SARS-CoV-2 BNT162b2 vaccination.

A 75-year-old man was vaccinated with SARS-CoV-2 on day 0 and day 23. From day 25, pustules began to appear.

A skin biopsy was performed. Tissue culture revealed the presence of *Trichophyton rubrum*.

The patient was treated with topical luliconazole and 100 mg/day oral fosravuconazole for 84 days, after which the symptoms resolved.

## 1 Introduction

Tinea, more commonly known as ringworm, is one of the most prevalent dermatological diseases and is caused by *Trichophyton rubrum* [1]. Topical medications are typically sufficient for the treatment of Tinea [2]; however, because *T. rubrum* can form subcutaneous abscesses in immunosuppressed patients, oral antifungal medications may be indicated [3].

Psoriasis is considered an inflammatory disease with autoimmune involvement. In addition to topical agents, immunosuppressive drugs and biologics are used for treatment [4]. Systemic therapy for psoriasis is associated with increased susceptibility to infection [5]. Therefore, vaccines are thought to play an important role in controlling infection in individuals with psoriasis [6].

Although vaccination against coronaviruses in patients with psoriasis is safe, new onset or exacerbation of psoriasis after vaccination has been reported [[7], [8], [9]]. To the best of our knowledge, the exacerbation of tinea following coronavirus vaccination has not previously been reported.

Here, we report a patient with psoriasis who developed *T. rubrum*-caused deep dermatomycosis after SARS-CoV-2 vaccination with BNT162b2.

## 2 Case presentation

A 75-year-old Japanese man received a SARS-CoV-2 vaccination with BNT162b2 on day 0 and day 23.

His medical history included diabetes mellitus and psoriasis vulgaris. He was taking metformin hydrochloride, sitagliptin phosphate hydrate, apremilast, calcipotriol hydrate, and betamethasone dipropionate ointment topically. He has been taking sitagliptin for about 10 years.

Pustules began to appear on day 25, mainly on the lumbar region. The patient was referred to our hospital on day 86 because of the lack of improvement in his condition.

**Fig. 1:**
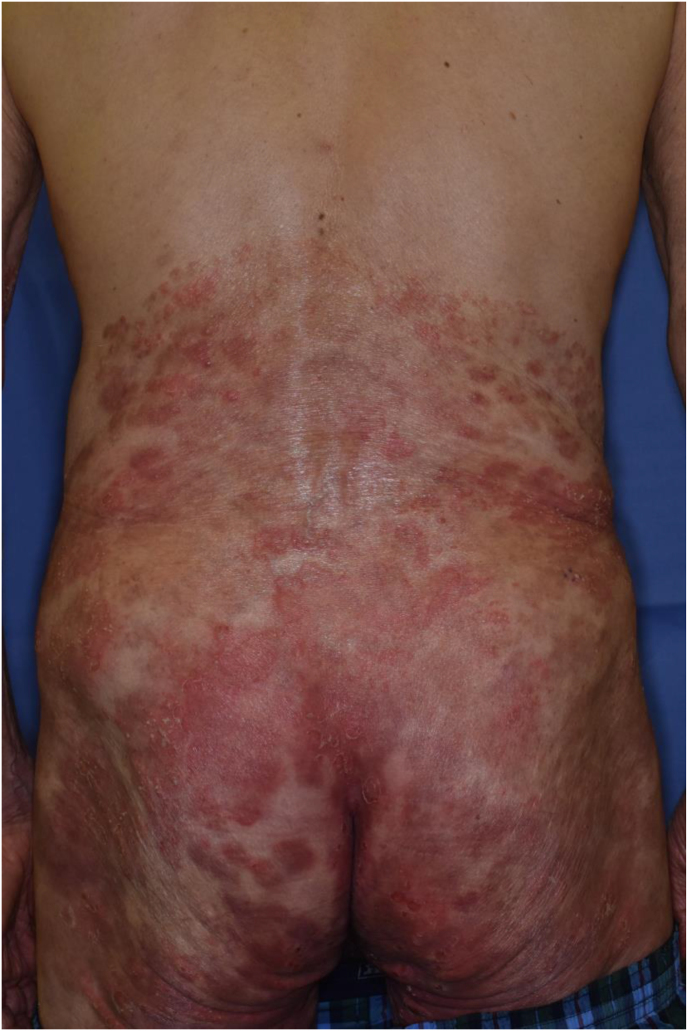
Clinical image of the back at initial examination.

Erythema with pustules was scattered mainly on the lumbar region and thighs (Fig. 1).

Blood tests at the first visit showed C-reactive protein levels of 0.16 mg/dL (normal range: 0 mg/dL–0.3mg/dL), an erythrocyte sedimentation rate of 14 mm (normal range: 2 mm–10 mm), white blood cell count of 9900/μL (normal range: 3300/μL–8600/μL), and β-D glucan of 23.7 pg/mL (normal range: 0 pg/mL–20 pg/mL). Skin biopsy was performed from the pustular area to differentiate pustular psoriasis and fungal infection. *Aspergillus* and *Cryptococcus* antigens were negative. KOH preparations were positive.

**Fig. 2:**
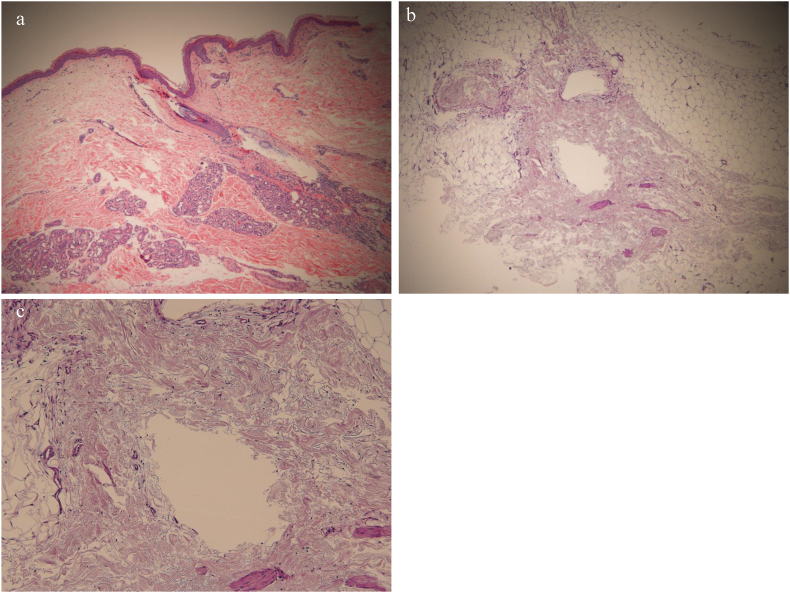
Skin biopsy. Hyperkeratosis was observed in the epidermis, and complex keratinization was observed in some areas. Inflammatory cell infiltration consisting of lymphocytes, plasma cells, and neutrophils was conspicuous in the epidermis and directly under the epidermis into the subcutaneous tissue. (a) Hematoxylin-eosin staining ( × 40), (b) Hematoxylin-eosin staining ( × 200), (c) Hematoxylin-eosin staining ( × 400).

Pathologically, the epidermis showed hyperkeratosis and some complex keratinization. Inflammatory cell infiltrates consisting of lymphocytes, plasma cells, and neutrophils were prominent in the epidermis and directly under the epidermis into the subcutaneous tissue (Fig. 2).

**Fig. 3:**
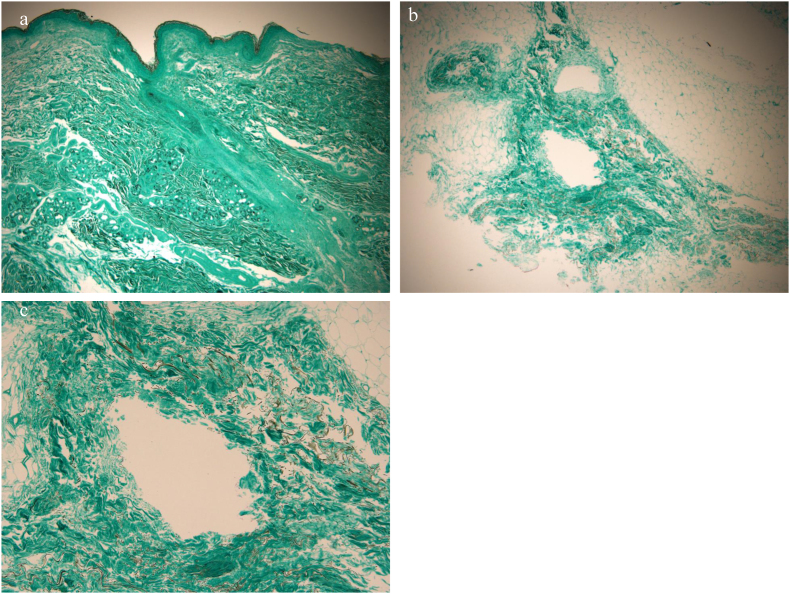
Skin biopsy. Many filamentous fungi were observed subcutaneously and in the stratum corneum. (a) Grocott staining ( × 40), (b) Grocott staining ( × 200), (c) Grocott staining ( × 400).

**Fig. 4:**
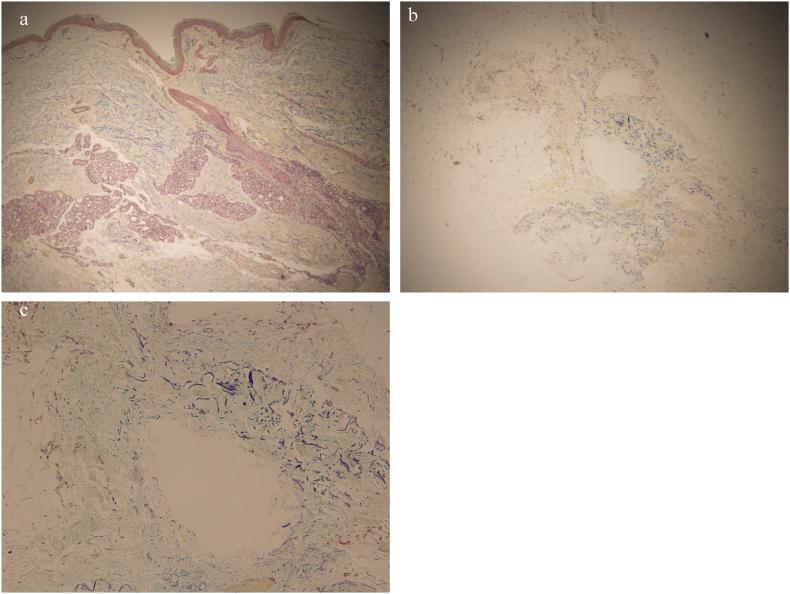
Skin biopsy. Many filamentous fungi were observed mainly in the stratum corneum but also subcutaneously. (a) Periodic acid-Schiff stain ( × 40), (b) Periodic acid-Schiff stain ( × 200), (c) Periodic acid-Schiff stain ( × 400).

Grocott staining and the Periodic acid-Schiff stain showed numerous filamentous fungi mainly in the stratum corneum but also subcutaneously (Fig. 3 and Fig. 4). Tissue culture revealed the presence of *T. rubrum* (MALDI-TOF/MS).

Based on the above tests, the patient was diagnosed with deep-seated dermatomycosis caused by *T. rubrum*. The patient was treated with topical luliconazole and 100 mg/day oral fosravuconazole for 84 days, after which the symptoms resolved, and β-D glucan became negative.

## 3 Discussion

The present report described a case of deep dermatomycosis caused by *T. rubrum*, which was thought to have worsened after SARS-CoV-2 vaccination with BNT162b2.

Because *T. rubrum* infection has as a pustular psoriasis-like clinical appearance with the use of IL17 inhibitor and immunosuppressive drugs, it is important to differentiate pustular psoriasis from *T. rubrum* infection [[10], [11], [12]]. The clinical symptoms in this case also resembled those of pustular psoriasis, which is consistent with previous reports.

On the other hand, this case differed from previous reports in that the patient had diabetes mellitus but was not immunosuppressed. Diabetes mellitus is known to be associated with dermatophyte mycosis. Although it cannot be denied that the patient was susceptible to severe disease due to diabetes, it is difficult to think that it triggered the severe disease. It is also known that diabetes medication also has an effect on vaginal candida, but in this case, the diabetes medication had not been changed for more than 10 years, so it is difficult to believe that it was the trigger for this severe disease [13].

It is known that vaccination against coronaviruses sometimes results in elevation of various cytokines [7]. We hypothesized that the coronavirus vaccine increased serum IL-6, which in turn increased serum ferritin levels, thereby creating an environment conducive to the development of fungal infections.

Fosravuconazole is a drug that has recently come into use in Japan as a treatment for onychomycosis [14]. The efficacy of fosravuconazole for the treatment of onychomycosis as well as other sites has been reported in recent years, but to our knowledge, this is the first report of its efficacy against deep-seated dermatomycosis [15].

Also, to the best of our knowledge, this is the first report of the occurrence of deep-seated dermatomycosis after SARS-CoV-2 vaccination.

In this case, we were able to appropriately diagnose and treat a deep dermatomycosis requiring differentiation in a situation where SARS-CoV-2 vaccination with BNT162b2 was reported to exacerbate psoriasis [[9], [10], [11], [12]]. Therefore, physicians should consider the occurrence of deep dermatomycosis after SARS-CoV-2 vaccination with BNT162b2.

## Conflict of interest

None.

## Ethical form

This research did not receive any specific grant from funding agencies in the public, commercial, or not-for-profit sectors. The authors declare no conflicts of interest. Written and signed consent to publish the case report was obtained from the patient.

## Consent

Please declare that you have obtained written and signed consent to publish the case report from the patient or legal guardian(s). Please state that consent has been obtained from the patient or legal guardian(s) Written informed consent was obtained from the patient or legal guardian(s) for publication of this case report and accompanying images. A copy of the written consent is available for review by the Editor-in-Chief of this journal on request.

## Declaration of competing interest

Please declare any financial or personal interests that might be potentially viewed to influence the work presented. Interests could include consultancies, honoraria, patent ownership or other. If there are none state ‘there are none’. Please state any competing interestsNone.

## References

[1] Ilkit M., Durdu M. (2015). Tinea pedis: the etiology and global epidemiology of a common fungal infection. Crit. Rev. Microbiol..

[2] Crawford F., Hollis S. (2007). Topical treatments for fungal infections of the skin and nails of the foot. Cochrane Database Syst. Rev..

[3] Toussaint F., Sticherling M. (2019). Multiple dermal abscesses by *Trichophyton rubrum* in an immunocompromised patient. Front. Med..

[4] Boehncke W.H., Schön M.P. (2015). Psoriasis, Lancet.

[5] Wakkee M., de Vries E., van den Haak P., Nijsten T. (2011). Increased risk of infectious disease requiring hospitalization among patients with psoriasis: a population-based cohort. J. Am. Acad. Dermatol..

[6] Chiricozzi A., Gisondi P., Bellinato F., Girolomoni G. (2020). Immune response to vaccination in patients with psoriasis treated with systemic therapies. Vaccines.

[7] Talamonti M., Galluzzo M. (2021). Safety of COVID-19 vaccines in patients with psoriasis undergoing therapy with anti-interleukin agents. Expet Opin. Biol. Ther..

[8] Pacifico A., d'Arino A., Pigatto P.D.M., Malagoli P., Damiani G., Young Dermatologists Italian Network (2021). COVID-19 vaccines do not trigger psoriasis flares in patients with psoriasis treated with apremilast. Clin. Exp. Dermatol..

[9] Elamin S., Hinds F., Tolland J. (2021). A case of de novo generalised pustular psoriasis following Oxford-AstraZeneca COVID-19 Vaccine. Clin. Exp. Dermatol..

[10] Feily A., Namazi M.R., Seifmanesh H. (2011). Generalized pustular psoriasis-like dermatophytosis due to *Trichophyton rubrum*. Acta Dermatovenerol. Croat..

[11] Serarslan G. (2007). Pustular psoriasis-like tinea incognito due to *Trichophyton rubrum*. Mycoses.

[12] Emelianov V., Feldmeyer L., Yawalkar N., Heidemeyer K. (2021). Tineа Corporis with *Trichophyton Rubrum* mimicking a flare-up of psoriasis under treatment with IL17-inhibitor ixekizumab. Case Rep. Dermatol..

[13] Rodrigues C.F., Rodrigues M.E., Henriques M. (2019). Candida sp. infections in patients with diabetes mellitus. J. Clin. Med..

[14] Watanabe S., Tsubouchi I., Okubo A. (2018). Efficacy and safety of fosravuconazole L-lysine ethanolate, a novel oral triazole antifungal agent, for the treatment of onychomycosis: a multicenter, double-blind, randomized phase III study. J. Dermatol..

[15] Takeshima R., Asahina Y., Yaguchii T., Sato T. (2020). Tinea barbae due to Trichophyton rubrum successfully treated using oral fosravuconazole l-lysine ethanolate. J. Dermatol..

